# Corneal Higher-Order Aberrations and Posterior Segment Changes in Keratoconus: A Multimodal OCT and OCTA Study

**DOI:** 10.3390/diagnostics16081212

**Published:** 2026-04-18

**Authors:** Ayşe Tüfekçi Balıkçı, Özlem Candan, Ayşe Burcu, Nurten Ünlü

**Affiliations:** Department of Ophthalmology, Ankara Training and Research Hospital, University of Health Sciences Turkey, Ankara 06230, Turkey; ozlem_aydnoglu@hotmail.com (Ö.C.); anurozler@yahoo.com.tr (A.B.); unlunurten@yahoo.com (N.Ü.)

**Keywords:** keratoconus, higher-order aberration, optical coherence tomography, optical coherence tomography angiography

## Abstract

**Background/Objectives:** To evaluate the associations between corneal topographic irregularity, higher-order aberrations (HOAs), and posterior segment structural and microvascular parameters in keratoconus using optical coherence tomography (OCT) and OCT angiography (OCTA). **Methods:** In this cross-sectional study, 81 eyes with keratoconus and 60 healthy control eyes underwent corneal topography and wavefront analysis, spectral-domain OCT with enhanced depth imaging, and OCTA. Retinal layer thicknesses, choroidal thickness and area metrics, choroidal vascularity index (CVI), and OCTA-derived vascular parameters were analyzed. Associations were assessed using Spearman correlation analysis with false discovery rate (FDR) correction. **Results:** Compared with controls, keratoconus eyes showed significantly increased corneal curvature, corneal irregularity indices, and HOAs (all *p* < 0.001). Structural OCT analysis demonstrated preserved inner retinal layers, whereas outer nuclear layer thickness was reduced (*p* < 0.001) and overall outer retinal layer thickness was increased (*p* = 0.005). Choroidal thickness and both total and luminal choroidal areas were significantly greater in keratoconus eyes (all *p* ≤ 0.011), while CVI did not differ between groups (*p* > 0.05). OCTA revealed reduced superficial capillary plexus vessel density at the whole image and perifoveal regions (all *p* < 0.001), whereas deep capillary plexus and foveal avascular zone metrics were largely preserved. Correlation analyses identified only weak and inconsistent associations between corneal parameters, HOAs, and posterior segment measurements, none of which remained statistically significant after FDR correction. **Conclusions:** Despite pronounced anterior segment deformation and optical degradation, posterior segment structural and microvascular alterations in keratoconus are limited and weakly related to corneal disease severity. These findings support a predominantly anterior segment centered pathophysiology of keratoconus and highlight the importance of stringent multiple-comparison control in multimodal imaging studies.

## 1. Introduction

Keratoconus (KC) is a progressive, non-inflammatory corneal ectatic disorder characterized by thinning and protrusion of the central or paracentral stroma, leading to irregular astigmatism, decreased visual acuity, and increased higher-order aberrations (HOAs), all of which impair visual function and quality of life [1]. Although both genetic and environmental factors contribute to its pathogenesis, the exact mechanisms remain incompletely understood [2]. Traditionally considered an anterior segment disease, KC has been increasingly associated with posterior segment alterations. Studies have reported changes in the retinal nerve fiber layer (RNFL), increased macular and peripapillary choroidal thickness, and macular dysfunction detected via multifocal electroretinography (mfERG) [3,4]. With the advent of advanced imaging modalities, these posterior segment changes can now be evaluated in greater detail. Optical coherence tomography (OCT) provides high-resolution cross-sectional imaging of retinal and choroidal structures, enabling precise assessment of layer-specific thickness and morphology. In contrast, optical coherence tomography angiography (OCTA) is a non-invasive technique that detects motion contrast from erythrocytes to visualize retinal and choroidal microvasculature without the need for dye injection. Using OCTA, reduced vascular density in macular and peripapillary regions has been demonstrated in KC, suggesting potential microvascular involvement [5,6].

One of the hallmark features of KC is the presence of significant ocular aberrations. These aberrations, particularly HOAs, can cause notable visual degradation even in the early stages of the disease, depending on the location and severity of the ectasia. While some level of HOAs is present in all eyes, keratoconic eyes typically exhibit five to six times higher HOA levels compared to healthy eyes. Both low- and high-order aberrations are strongly correlated with vision-related quality of life [7].

Although previous studies have independently evaluated corneal topographic abnormalities, HOAs, and posterior segment findings in KC, the relationship between these parameters remains incompletely understood. In particular, there is a lack of comprehensive studies integrating anterior segment optical quality metrics with both structural and microvascular posterior segment changes using multimodal imaging approaches.

Therefore, the aim of this study is to investigate the associations between HOAs and corneal topographic parameters with structural retinal changes assessed by OCT, as well as microvascular alterations evaluated by OCTA in patients with KC. By combining these modalities, this study provides a more integrated and comprehensive understanding of the interplay between anterior and posterior segment alterations in keratoconus.

## 2. Materials and Methods

This cross-sectional study included 81 eyes of 81 patients with KC and 60 eyes of 60 age- and sex-matched healthy controls, all of whom were recruited from University of Health Sciences, Ankara Training and Research Hospital, Ankara, Turkey. Written informed consent was obtained from all participants. This study was conducted in accordance with the tenets of the Declaration of Helsinki and was approved by the local ethics committee (approval no. E-25-601).

Eligible KC patients were aged 18–45 years and had stage 1–3 disease based on the Amsler–Krumeich classification with adequate fixation for imaging. Exclusion criteria included other ocular diseases, systemic disorders with ocular involvement, previous ocular surgery or trauma, axial length >25.0 mm, chronic medication use, and contact lens wear within 3 weeks for rigid lenses or 1 week for soft lenses.

Controls had no history of ocular disease and included emmetropic (manifest refraction spherical equivalent [MRSE] ± 0.50 D) and mildly ametropic eyes (myopia ≤ −3.50 D and astigmatism ≤ ±1.00 D), all with corrected distance visual acuity of 20/20 or better and with no clinical or tomographic signs of keratoconus.

All participants underwent a comprehensive ophthalmologic examination including visual acuity testing, slit-lamp biomicroscopy, fundus examination, intraocular pressure measurement, corneal topography, spectral-domain OCT, and OCTA imaging. Posterior segment scans were obtained following pupil dilation with 1% tropicamide (Polpharma, Warsaw, Poland).

### 2.1. Corneal Topography and Aberration Analysis

Corneal topography and wavefront aberrometry were performed using a Scheimpflug–Placido-based imaging system (Sirius, Phoenix software v.2.6.4.44, Costruzione Strumenti Oftalmici, Florence, Italy). For each eye, three consecutive measurements aligned to the visual axis were acquired, and only high-quality scans labeled as “OK” were included. Patients were instructed to blink before each capture to ensure tear film stability.

Measurements were obtained for both 3.0 mm and 6.0 mm simulated pupil diameters. Total ocular, anterior corneal, and internal optical aberrations were evaluated using Zernike polynomial decomposition up to the 7th order. Root mean square (RMS) values were calculated for total aberrations, HOAs, coma [Z(3, ±1)], trefoil [Z(3, ±3)], spherical aberration [Z(4, 0)], and Zernike-derived astigmatism [Z(2, ±2)]. Additional corneal topographic parameters included anterior and posterior mean keratometry (Kmean) and maximum keratometry (Kmax), central and thinnest corneal thickness (CCT, TCT), thinnest point location, symmetry indices (SIf, SIb), central–surrounding indices (CSIf, CSIb), and ectasia indices (EIf, EIb), defined according to standard criteria for keratoconus screening and assessment of corneal regularity.

Optical quality was further assessed using optical path difference (OPD) and Strehl ratio (SR) metrics derived from aberrometric analysis. OPD was used to quantify cumulative wavefront deviations across the optical system, while the Strehl ratio was calculated as the ratio of the measured point spread function peak intensity to that of an ideal diffraction-limited system, with values closer to 1.0 indicating better optical quality.

### 2.2. Posterior Segment Imaging

Posterior segment imaging was performed using spectral-domain optical coherence tomography (SD-OCT; Heidelberg Engineering, Heidelberg, Germany) and optical coherence tomography angiography (OCTA; RTVue XR Avanti, Optovue Inc., Fremont, CA, USA). All scans were acquired under standardized lighting conditions between 9:00 and 11:00 a.m. to minimize diurnal variation. Only high-quality images with a signal strength index ≥7/10 and without motion or segmentation artifacts were included. OCT- and OCTA-derived parameters were analyzed using the manufacturers’ integrated software and correlated with anterior segment topography and wavefront aberrometry metrics.

### 2.3. OCT Imaging

Retinal structural parameters were assessed using SD-OCT. Central macular thickness (CMT) was defined as the mean retinal thickness within the central 1-mm diameter circle centered on the fovea. Parafoveal retinal thickness was obtained from the superior, inferior, nasal, and temporal quadrants of the parafoveal ring. Automated segmentation was used to quantify individual retinal layers, including the ganglion cell layer (GCL), inner plexiform layer (IPL), inner nuclear layer (INL), outer plexiform layer (OPL), outer nuclear layer (ONL), retinal pigment epithelium (RPE), inner retinal layers (IRLs), and outer retinal layers (ORLs). In this study, the inner retinal layers (IRLs) were defined as the layers extending from the internal limiting membrane (ILM) to the INL, including the GCL and IPL. The ORLs were defined as the layers extending from the OPL to the RPE, including the ONL and photoreceptor layers. Segmentation accuracy was reviewed manually. Thickness measurements were analyzed separately for central and parafoveal regions.

Choroidal parameters were evaluated using enhanced depth imaging OCT (EDI-OCT). Subfoveal choroidal thickness (SFCT) was measured manually as the perpendicular distance between the outer border of the RPE and the choroid–sclera interface. Nasal and temporal choroidal thicknesses relative to the fovea were also measured on horizontal and vertical EDI-OCT scans. Choroidal vascular parameters, including luminal choroidal area (LCA), total choroidal area (TCA), and choroidal vascularity index (CVI), were quantified using ImageJ software (version 1.54r, National Institutes of Health, Bethesda, MD, USA). CVI was calculated as the ratio of LCA to TCA (CVI = LCA/TCA). Images were converted to 8-bit format, and a standardized region extending 750 μm nasally and temporally from the foveal center was manually delineated. Image binarization was performed using the Niblack local thresholding method to differentiate luminal and stromal components. All manual measurements were performed by the same experienced examiner.

### 2.4. OCTA Imaging

Macular microvasculature was evaluated using OCTA. Two 6 × 6 mm scans centered on the fovea were acquired for each eye, and the scan with the higher signal strength was used for analysis. Vascular density (VD) parameters of the superficial capillary plexus (SCP) and deep capillary plexus (DCP), as well as the foveal avascular zone (FAZ) area, were automatically calculated using AngioAnalytics™ software, RTVue XR Avanti system (Optovue Inc., Fremont, CA, USA; Optovue, RTVueXR Avanti Optovue Version 2017.1.0.151). VD was defined as the percentage of the analyzed area occupied by flow pixels exceeding the noise threshold. Measurements were analyzed according to the Early Treatment Diabetic Retinopathy Study (ETDRS) grid, including central (1 mm), parafoveal (1–3 mm), perifoveal (3–6 mm), and whole scan regions. FAZ area, non-flow area (NFA), and choriocapillaris flow void area (CCPFA) were recorded. All OCTA-derived measurements were reviewed for accuracy by an experienced examiner.

### 2.5. Statistical Analysis

All statistical analyses were performed using IBM SPSS Statistics version 23.0 (IBM Corp., Armonk, NY, USA). Continuous variables were expressed as mean ± standard deviation or median (interquartile range), as appropriate. Age and continuous clinical parameters were compared between the KC and control groups using the Mann–Whitney U test, while sex distribution was compared using Pearson’s chi-square test. Corneal topography parameters, higher-order aberrations, and OCT/OCTA measurements were compared between groups using the Mann–Whitney U test. Associations between corneal topography and higher-order aberration parameters and OCT/OCTA measurements were evaluated exclusively in the keratoconus group using Spearman’s rank correlation analysis. To account for multiple comparisons, false discovery rate (FDR) correction was applied using the Benjamini–Hochberg procedure. Correlation results were summarized using heatmap visualization, and complete correlation data are provided in the Supplementary Materials. A two-tailed *p* value < 0.05 and FDR-adjusted *p* value < 0.05 were considered statistically significant. To enhance interpretability, selected representative parameters reflecting the main outcomes were additionally presented using graphical visualizations (e.g., boxplots), allowing clearer comparison of group differences and data distribution.

## 3. Results

A total of 141 participants were included in the study, comprising 81 patients with KC and 60 control subjects. The KC group consisted of 40 females (49.4%) and 41 males (50.6%), while the control group included 32 females (53.3%) and 28 males (46.7%). There was no statistically significant difference in sex distribution between the groups (Pearson’s chi-square test, *p* = 0.643). The mean age was 27.73 ± 6.85 years in the KC group and 26.60 ± 6.72 years in the control group. Age distribution did not differ significantly between the two groups (independent samples *t*-test, *p* = 0.331). Best-corrected visual acuity (BCVA) was significantly worse in the KC group compared with controls (*p* < 0.001, Mann–Whitney U test). Axial length was comparable between the KC and control groups (23.98 ± 0.51 mm vs. 24.15 ± 0.46 mm, *p* > 0.05). Refractive error analysis showed significantly higher myopia and astigmatism values in the keratoconus group than in the control group (both *p* < 0.001). Similarly, topographic astigmatism was significantly greater in eyes with keratoconus compared to controls (*p* < 0.001). According to the Amsler–Krumeich classification, 70 eyes (86.4%) were classified as stage 1, 10 eyes (12.3%) as stage 2, and only 1 eye (1.2%) as stage 3 in the KC group.

Additional sex-stratified analyses were performed to evaluate potential sex-related effects. In the keratoconus group, no significant differences were observed between male and female participants in key parameters, including HOA6mm (*p* = 0.767) and Kmax (*p* = 0.167). Although a statistically significant difference was noted for INL thickness (*p* = 0.027), this finding was isolated and not supported by other posterior segment parameters. In the control group, certain anatomical parameters, including Kmax (*p* = 0.012), TCT (*p* = 0.036), and INL thickness (*p* = 0.004), differed between sexes. However, no significant differences were observed in HOA values (*p* = 0.963) or OCTA-derived vascular parameters (all *p* > 0.05). Overall, sex did not significantly influence the primary outcomes or the observed relationships between corneal and posterior segment parameters.

Corneal topography and HOA parameters differed significantly between KC and control groups (Table 1). Eyes with KC demonstrated significantly steeper anterior and posterior corneal curvature values, including higher Kmean and Kmax measurements, along with reduced central and thinnest corneal thickness parameters (all *p* < 0.001). Indices reflecting corneal irregularity and elevation were consistently increased in the KC group. HOA analysis revealed significantly increased aberrations across both 3-mm and 6-mm optical zones, accompanied by poorer optical quality metrics compared with controls.

**Figure 1:**
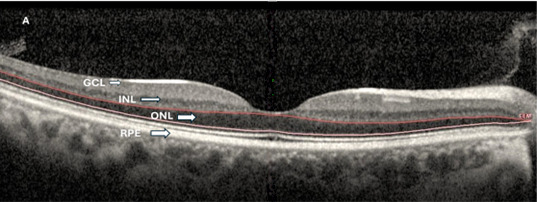

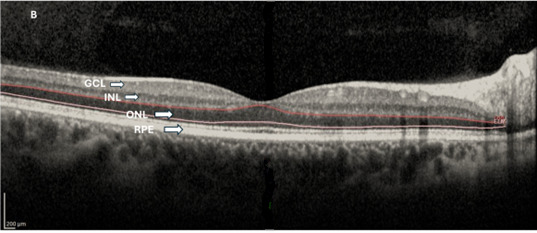
Representative OCT B-scan images with retinal layer segmentation from a healthy control (**A**) and a keratoconus eye (**B**). Major retinal layers, including the ganglion cell layer (GCL), inner nuclear layer (INL), outer nuclear layer (ONL), and retinal pigment epithelium (RPE), are indicated for illustrative purposes. While no obvious qualitative differences are apparent between groups, quantitative analysis revealed a significant reduction in ONL thickness in keratoconus eyes.

Structural OCT analysis revealed selective layer-specific differences between groups (Table 2). Inner retinal layer thicknesses, including the GCL, IPL, INL, and IRL thickness, did not differ significantly between KC and control eyes. In contrast, ONL thickness was significantly reduced in the KC group (*p* < 0.001), while overall ORL thickness was significantly increased (*p* = 0.005). Central macular thickness and parafoveal thickness measurements were comparable between groups (Figure 1).

**Figure 2:**
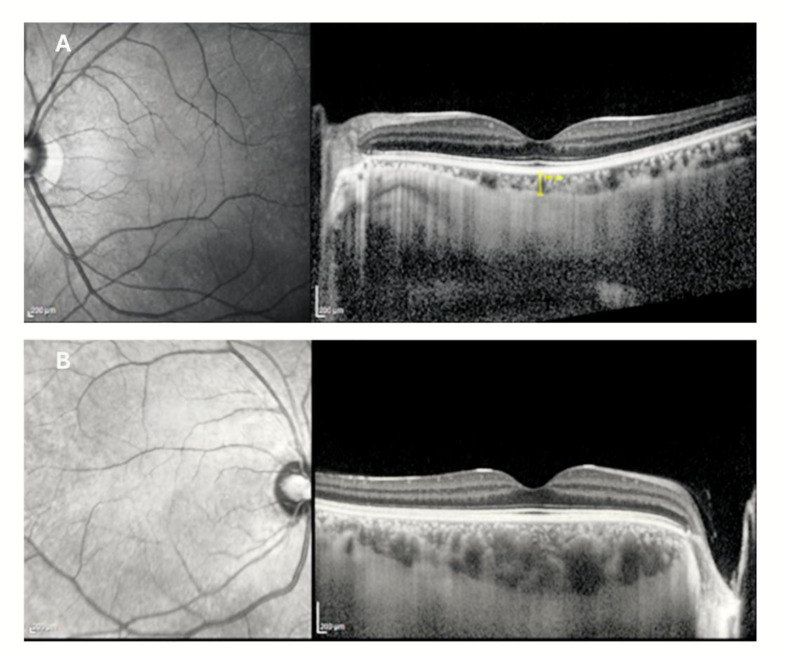
Representative enhanced depth imaging optical coherence tomography (EDI-OCT) images from a healthy control eye (**A**) and a keratoconus eye (**B**). Subfoveal choroidal thickness is measured in both images. Compared to the control, the keratoconus eye demonstrates increased choroidal thickness, consistent with the quantitative analysis.

Choroidal assessment demonstrated significantly increased temporal, subfoveal, and nasal choroidal thicknesses in KC eyes (all *p* ≤ 0.011) (Figure 2). TCA and LCA were also significantly higher in the KC group, whereas the CVI did not differ significantly between groups.

**Figure 3:**
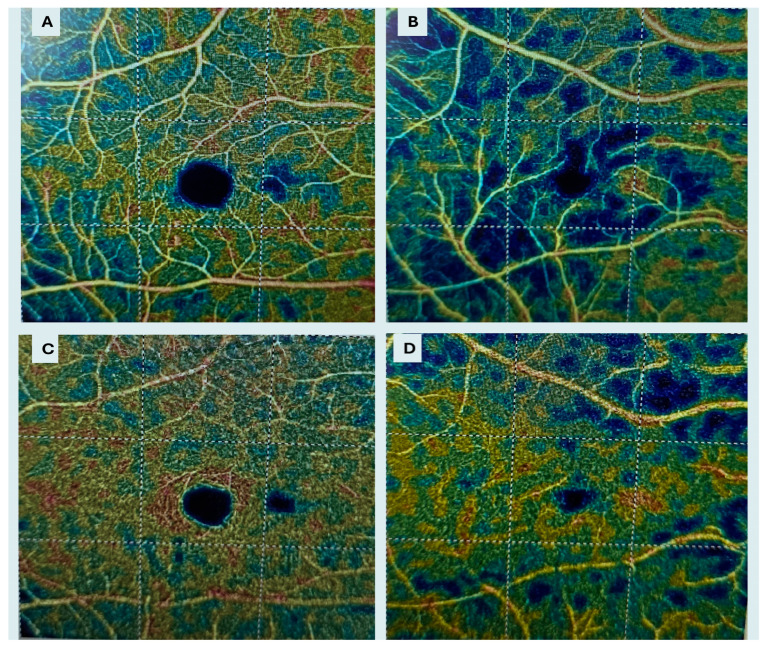
Representative optical coherence tomography angiography (OCTA) images of the superficial capillary plexus (SCP) and deep capillary plexus (DCP) in a healthy control (**A**,**C**) and a keratoconus eye (**B**,**D**). Compared to controls, keratoconus eyes demonstrate reduced vessel density and increased heterogeneity, particularly in the SCP. In the DCP, alterations are less pronounced, with only subtle reductions observed, consistent with the quantitative analysis.

OCTA analysis showed reduced SCP VD in KC eyes at the whole image, parafoveal, and perifoveal regions (all *p* < 0.001). DCP VD parameters were largely similar between groups, except for a modest reduction in perifoveal DCP vessel density in the KC group (*p* = 0.031). FAZ area did not differ significantly between groups (Figure 3). NFA and CCPFA were significantly increased in KC eyes (*p* = 0.038 and *p* = 0.010, respectively).

**Figure 4:**
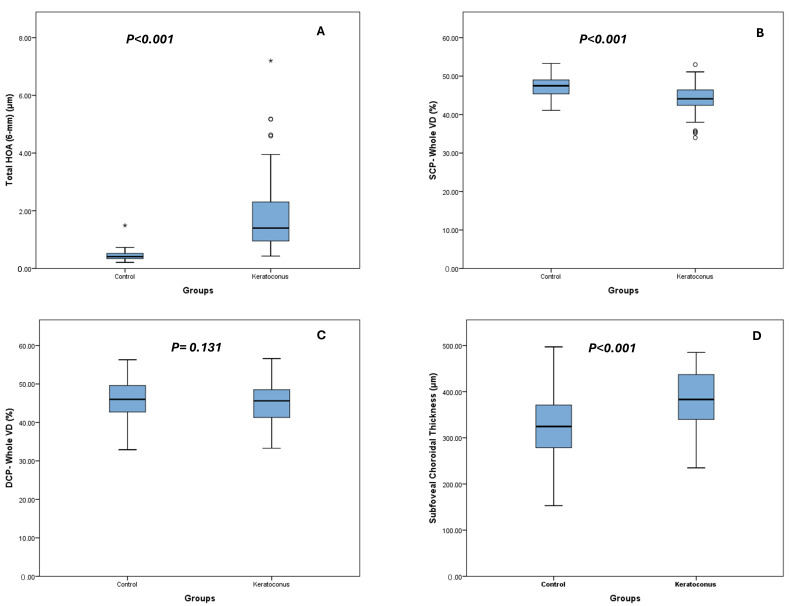
Anterior optical degradation and selective posterior segment alterations in keratoconus. Boxplots comparing (**A**) total higher-order aberrations (6-mm zone), (**B**) superficial capillary plexus vessel density, (**C**) deep capillary plexus vessel density, and (**D**) subfoveal choroidal thickness between keratoconus and control groups. Keratoconus was associated with markedly increased HOAs and choroidal thickening, accompanied by reduced SCP vessel density, while DCP parameters remained unchanged, indicating a non-uniform pattern of posterior segment involvement. *p*-values are indicated for each comparison. In the boxplot, circles (○) indicate outliers and asterisks (*) represent extreme outliers, as defined by SPSS.

Selected parameters, including higher-order aberrations (HOAs), subfoveal choroidal thickness, and vessel density metrics (SCP and DCP), are graphically illustrated in Figure 4 to facilitate visual comparison between groups.

Structural OCT analysis revealed several weak to moderate correlations between corneal topography and HOA parameters and selected retinal and choroidal measurements (Table 3). Corneal curvature and irregularity indices showed a tendency toward associations with inner retinal layer thicknesses, whereas correlations with outer retinal layers were limited. However, none of these associations remained statistically significant after FDR adjustment for multiple comparisons. A comprehensive correlation analysis including all corneal and OCT parameters is provided in Supplementary Table S1.

In contrast, correlations between corneal topography and HOA parameters and OCTA-derived vascular metrics were uniformly weak (Table 4). Although isolated associations were observed at the nominal significance level, none of the correlations with SCP or DCP VD or FAZ measurements remained statistically significant after FDR correction. Detailed correlation matrices are provided in the Supplementary Tables.

**Figure 5:**
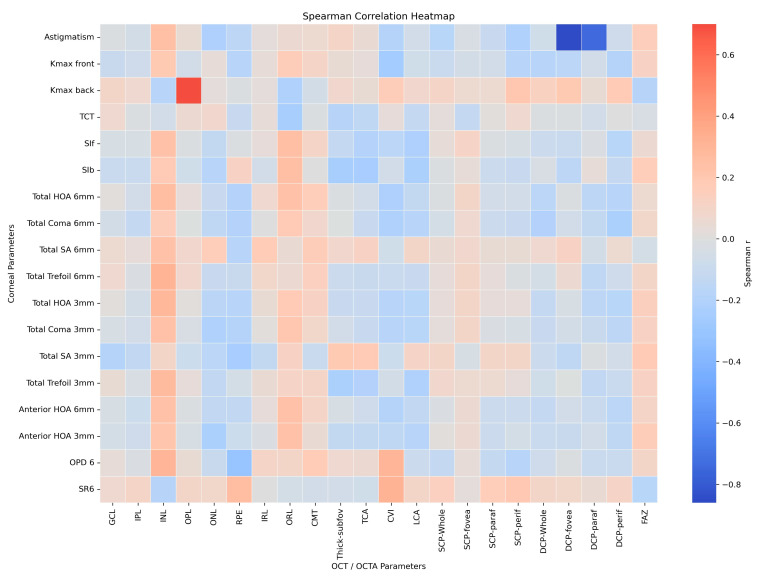
Heatmap showing correlations between corneal higher-order aberrations and posterior segment parameters in keratoconus. The heatmap illustrates Spearman correlation coefficients between corneal higher-order aberrations and posterior segment structural and microvascular measurements obtained by optical coherence tomography and OCT angiography. Color intensity represents the strength and direction of correlations, with warmer colors indicating positive correlations and cooler colors indicating negative correlations. Only correlations passing the predefined significance threshold are displayed.

An illustrative heatmap based on selected parameters is provided in Figure 5 to visually summarize these relationships.

Supplementary analyses including the complete set of corneal topography, aberration, OCT, and OCTA parameters confirmed the robustness of the primary findings. Expanded correlation matrices demonstrated consistent patterns, with structural OCT parameters showing more frequent associations with corneal metrics than OCTA-derived vascular parameters. No additional significant correlations were identified after FDR adjustment.

## 4. Discussion

Keratoconus has traditionally been regarded as an anterior segment disorder; however, an increasing number of OCT and OCTA studies have raised questions regarding potential posterior segment involvement. While several investigations have reported alterations in retinal thickness, choroidal morphology, or macular and peripapillary vascular density, the reported findings remain heterogeneous and sometimes contradictory [5,6,8,9,10]. Against this background, the present study provides a comprehensive multimodal evaluation of the relationship between corneal topographic irregularity, higher-order aberrations, and posterior segment structural and microvascular parameters in KC. Our results indicate that, despite marked corneal deformation and optical degradation, posterior segment alterations are selective, heterogeneous, and only weakly associated with corneal disease severity.

Consistent with previous topography and wavefront studies, KC eyes in the present cohort demonstrated pronounced abnormalities in corneal curvature, pachymetry, elevation indices, and HOAs, with a predominance of coma-related aberrations and reduced Strehl ratio. These findings are in line with earlier reports describing substantial optical quality degradation as a hallmark of keratoconic biomechanics [11,12]. Importantly, several authors have suggested that chronic optical blur may influence OCT-based retinal measurements, an issue that must be considered when interpreting posterior segment findings in KC [13,14].

Structural OCT analysis revealed relatively preserved inner retinal layers, including the GCL and INL, in keratoconus eyes. In contrast, selective alterations were observed in outer retinal and choroidal parameters, including increased choroidal thickness and area measurements. Previous OCT studies have reported conflicting results regarding retinal thickness in KC with some demonstrating no difference compared to controls, others reporting thinning of the retinal nerve fiber layer or GCL, and several describing increased macular or posterior pole thickness [3,4,9,15,16,17]. In line with studies showing preserved macular thickness in early or mild KC [16], the present study found similar central and perifoveal macular thickness values between KC patients and controls, supporting the concept that macular structure remains largely unaffected in the absence of advanced disease. More recent segmentation-based analyses have suggested that KC-related retinal changes may preferentially involve the INL and ORLs [18,19]. In this context, the observed reduction in ONL thickness alongside a relative increase in overall ORL thickness may appear contradictory at first glance. ONL thinning is generally interpreted as a marker of photoreceptor loss or dysfunction. This interpretation is supported by previous segmentation-based studies reporting preferential involvement of outer retinal layers in keratoconus, particularly thinning of the ONL and RPE [18,19]. However, changes in overall outer retinal thickness may not parallel ONL alterations, as different outer retinal components—including photoreceptor inner and outer segments and the retinal pigment epithelium—may be affected differently.

Importantly, previous studies have reported inconsistent findings regarding outer retinal layer changes in keratoconus, with some demonstrating thinning, others reporting regional thickening, and some finding no significant differences [10]. These discrepancies may be attributed to differences in disease stage, imaging protocols, and segmentation approaches. In this context, the coexistence of ONL thinning and increased ORL thickness in our study may reflect the heterogeneous and multifactorial nature of retinal alterations in keratoconus rather than a uniform structural response. In addition, the increased choroidal thickness observed in our cohort may have contributed to outer retinal changes through choroid–retina interactions, potentially influencing outer retinal structure and metabolism. Previous OCT-based studies have reported increased INL thickness in more advanced KC, which has been hypothesized to reflect Müller cell activation in response to chronic oxidative stress [19]. Although inner retinal layers were largely preserved in our cohort, the weak association observed between HOA and INL thickness may partially align with these observations; however, this finding should be interpreted with caution and does not provide direct evidence of neuroglial involvement. However, this association was weak and should be interpreted cautiously. Keratoconus has been associated with systemic inflammation and oxidative stress, which may contribute to retinal structural alterations. Previous studies have suggested that oxidative stress-related mechanisms may affect photoreceptors and retinal pigment epithelium cells, potentially leading to outer retinal changes [19]. In addition, given that the choroid provides oxygen and metabolic support to the outer retina, alterations in choroidal structure may further influence outer retinal integrity. These mechanisms, together with disease stage-related variability and methodological differences, may help explain the heterogeneous retinal findings reported across studies.

Choroidal findings in KC have also varied considerably across studies. Increased subfoveal choroidal thickness and enlarged choroidal area have been reported by several authors, often accompanied by elevated CVI values [6,10,20,21,22]. In contrast, our study demonstrated increased choroidal thickness and enlargement of TCA and LCA without a corresponding change in CVI. Taken together with structural changes observed in this cohort, our findings may indicate that choroidal alterations in KC primarily reflect volumetric expansion rather than redistribution between vascular and stromal components. Similar findings have been suggested in the literature, where choroidal changes have been attributed to connective tissue alterations and extracellular matrix remodeling that may extend beyond the cornea to involve other ocular structures [10]. The absence of significant correlations between choroidal parameters and corneal topographic or aberration metrics further supports the notion that choroidal changes do not scale linearly with ectatic severity. Collectively, these findings suggest that structural and microvascular posterior segment alterations in keratoconus may follow partially independent patterns.

With respect to macular microvasculature, prior OCTA studies have reported reduced superficial and deep capillary plexus densities, particularly at the macular and peripapillary levels, whereas others have found preserved or even increased flow-related parameters [5,6,8,17,23,24]. In the present study, OCTA revealed a selective reduction in SCP VD, while DCP and FAZ metrics remained largely preserved. Importantly, no robust associations between OCTA-derived flow parameters and corneal topography or HOAs were identified after false discovery rate correction. This observation aligns with previous reports describing weak or inconsistent correlations between corneal parameters and macular vascular density, suggesting relative independence of posterior segment vascular regulation from anterior segment deformation [17]. Alternatively, previously reported reductions in vascular density may be partly related to oxidative stress-induced microvascular changes or measurement variability in eyes with increased optical aberrations.

Differences between studies may be partly explained by variation in disease stage distribution, image quality, and analytical methodology [5]. Advanced KC has been associated with more pronounced OCTA abnormalities, but it is also characterized by greater corneal irregularity and HOAs, which may compromise OCTA signal quality and segmentation accuracy [5,6]. By restricting analyses to high-quality scans and applying FDR correction, the present study provides a conservative assessment of posterior segment involvement, indicating that some previously reported associations may reflect methodological artifacts rather than consistent biological effects.

An additional observation of the present study is the differential behavior of structural and flow-based vascular metrics. Our findings suggest that the CVI and LCA primarily represent vascular architecture rather than dynamic blood flow, which may explain their closer association with OCT-derived parameters compared with OCTA flow metrics. These observations may be compatible with choroidal volumetric expansion rather than a primary shift in vascular–stromal balance.

Correlation analyses revealed only modest and inconsistent associations between corneal parameters, HOAs, and posterior segment measurements, none of which remained significant after adjustment for multiple comparisons. Although unadjusted analyses suggested trends involving RMS-based aberration metrics and selected retinal or choroidal parameters, these findings should be interpreted with caution. By contrast, some previous studies have reported moderate to strong negative correlations between macular SCP VDty and corneal severity indices, such as BAD-D scores and Kmax values, suggesting a potential association between corneal ectatic severity and macular microvascular alterations [5]. Taken together, these results suggest that any biomechanical or optical coupling between anterior and posterior segment alterations is likely weak and may be insufficient to result in clinically meaningful posterior segment impairment.

To our knowledge, this study represents one of the first comprehensive evaluations directly comparing corneal topography and aberration-based metrics with both OCT and OCTA derived posterior segment parameters under strict multiple-comparison control, thereby reducing the risk of spurious associations in high-dimensional imaging analyses.

Several limitations must be acknowledged. The cross-sectional design precludes causal inference, and the cohort predominantly comprised early-stage keratoconus, limiting conclusions regarding advanced disease. Moreover, OCTA measurements are inherently sensitive to image quality and segmentation accuracy, particularly in eyes with irregular optics. Accordingly, our findings should be interpreted as primarily representative of early to moderate keratoconus. Another limitation of this study is the predominance of early-stage keratoconus cases in our cohort, with the majority of eyes classified as stage 1 according to the Amsler–Krumeich classification. This distribution is partly attributable to image quality constraints inherent to OCTA acquisition, as reliable vascular measurements are more difficult to obtain in advanced keratoconus due to optical distortion and poor signal quality. Therefore, our findings primarily reflect early-stage disease characteristics and should be interpreted with caution when extrapolating to moderate and advanced stages.

Given the large number of correlation tests performed, FDR correction substantially attenuated nominal associations observed in unadjusted analyses. This discrepancy underscores the importance of stringent multiple-comparison control in high-dimensional imaging studies and suggests that previously reported associations should be interpreted cautiously as exploratory rather than definitive findings.

In summary, in agreement with the broader and heterogeneous OCT and OCTA literature, the present study demonstrates that marked anterior segment deformation and optical degradation in KC do not translate into robust or generalized posterior segment structural or microvascular compromise. Observed posterior segment changes appear to be subtle, stage-dependent, and likely reflect heterogeneous, multifactorial, and possibly subclinical processes rather than direct posterior extension of corneal ectatic pathology. By systematically comparing corneal topographic and HOA metrics with both structural OCT and OCTA parameters, and by applying FDR correction to account for multiple testing, the present study provides a more rigorous and integrated assessment of anterior–posterior segment relationships than previously reported. These findings reinforce a predominantly anterior segment centered understanding of keratoconus and highlight the importance of cautious interpretation of posterior segment associations in high-dimensional imaging studies.

## Figures and Tables

**Table 1:** Corneal topography and higher-order aberration parameters in keratoconus and control eyes.

Topography and Aberration Parameters	Control Group *N* = 60	Keratoconus Group *N* = 81	*p*-Value *
Astigmatism	0.79 ± 0.41	3.08 ± 1.86	<0.001
Pupil size (mm)	3.09 ± 0.33	3.42 ± 0.38	<0.001
Kmean front (diopters)	42.90 ± 1.25	46.04 ± 2.85	<0.001
Kmax front (diopters)	43.93 ± 1.42	51.07 ± 5.68	<0.001
Kmean back (diopters)	−6.02 ± 0.27	−6.56 ± 1.12	<0.001
Kmax back (diopters)	−6.30 ± 0.30	−8.18 ± 1.43	<0.001
CCT (μm)	533.24 ± 41.33	477.46 ± 42.71	<0.001
TCT (μm)	528.24 ± 40.73	459.28 ± 43.85	<0.001
Thinnest location (mm)	0.58 ± 0.26	0.88 ± 0.37	<0.001
SIf	0.12 ± 0.35	3.27 ± 2.86	<0.001
CSIf	0.56 ± 0.43	2.82 ± 3.08	<0.001
SIb	0.02 ± 0.06	0.94 ± 0.85	<0.001
CSIb	0.06 ± 0.08	0.72 ± 0.79	<0.001
EIf	2.45 ± 1.96	24.83 ± 16.91	<0.001
EIb	2.43 ± 1.80	52.92 ± 37.66	<0.001
Total RMS 6 mm	0.80 ±0.40	3.20 ± 1.72	<0.001
Total HOA 6 mm	0.44 ± 0.20	1.85 ± 1.29	<0.001
Total Astigmat 6 mm	0.62 ± 0.39	2.45 ± 1.44	<0.001
Total Coma 6 mm	0.25 ± 0.18	1.50 ± 1.17	<0.001
Total SA 6 mm	0.19 ± 0.09	0.26 ± 0.23	0.262
Total Trefoil 6 mm	0.18 ± 0.09	0.74 ± 0.44	<0.001
Total RMS 3 mm	0.21 ± 0.12	0.89 ± 0.63	<0.001
Total HOA 3 mm	0.10 ± 0.07	0.40 ± 0.37	<0.001
Total Astigmat 3 mm	0.18 ± 0.11	0.77 ± 0.54	<0.001
Total Coma 3 mm	0.04 ± 0.04	0.27 ± 0.33	<0.001
Total SA 3 mm	0.02 ± 0.01	0.07 ± 0.32	<0.001
Total Trefoil 3 mm	0.05 ± 0.04	0.21 ± 0.14	<0.001
Anterior RMS 6 mm	1.04 ± 0.44	3.89 ± 2.04	<0.001
Anterior HOA 6 mm	0.46 ± 0.17	2.33 ± 1.61	<0.001
Anterior Astigmat 6 mm	0.86 ± 0.48	2.91 ± 1.64	<0.001
Anterior Coma 6 mm	0.27 ± 0.17	1.93 ± 1.47	<0.001
Anterior SA 6 mm	0.21 ± 0.09	0.32 ± 0.29	0.146
Anterior Trefoil 6 mm	0.19 ± 0.09	0.83 ± 0.51	<0.001
Anterior RMS 3 mm	0.25 ± 0.12	1.09 ± 0.74	<0.001
Anterior HOA 3 mm	0.09 ± 0.05	0.49 ± 0.46	<0.001
Anterior Astigmat 3 mm	0.22 ± 0.12	0.93 ± 0.63	<0.001
Anterior Coma 3 mm	0.03 ± 0.02	0.41 ± 0.48	<0.001
Anterior SA 3 mm	0.01 ± 0.01	0.05 ± 0.05	<0.001
Anterior Trefoil 3 mm	0.04 ± 0.03	0.24 ± 0.17	<0.001
OPD 6 mm	0.96 ± 0.38	3.30 ± 1.73	<0.001
SR 6 mm	0.159 ± 0.05	0.071 ± 0.09	<0.001
OPD 3 mm	0.217 ± 0.11	0.90 ± 0.63	<0.001
SR 3 mm	0.390 ± 0.12	0.168 ± 0.10	<0.001

Kmean: mean keratometry; Kmax: maximum keratometry; CCT: central corneal thickness; TCT: thinnest corneal thickness; SIf: surface irregularity index (front); SIb: surface irregularity index (back); CSIf: central surface irregularity index (front); CSIb central surface irregularity index (back); EIf: elevation index (front); EIb: elevation index (back); RMS: root mean square; HOA: higher-order aberrations; SA: spherical aberration; OPD: optical path difference; SR: Strehl ratio. Values are presented as mean ± standard deviation. *p* < 0.05 is statistically significant. * Mann–Whitney U Test.

**Table 2:** Comparison of OCT and OCTA parameters between keratoconus and control groups.

OCT and OCTA Parameters	Control Group *N* = 60	Keratoconus Group *N* = 81	*p*-Value *
GCL (μm)	14.94 ± 5.04	16.22 ± 7.34	0.820
IPL (μm)	20.15 ± 3.99	20.74 ± 5.10	0.918
INL (μm)	18.60 ± 5.62	20.55 ± 9.05	0.398
OPL (μm)	25.53 ± 7.25	26.85 ± 7.35	0.237
ONL (μm)	91.86 ± 10.96	84.11 ± 12.23	<0.001
RPE (μm)	16.67 ± 1.44	16.91 ± 2.00	0.440
IRL (μm)	181.67 ± 20.93	179.65 ± 26.37	0.315
ORL (μm)	88.67 ± 3.67	90.80 ± 5.48	0.005
CMT (μm)	270.68 ± 21.05	271.40 ± 27.74	0.668
Parafovea Superior (μm)	345.67 ± 13.41	344.97 ± 14.79	0.964
Parafovea Inferior (μm)	343.60 ± 12.55	343.30 ± 13.62	0.804
Parafovea Temporal (μm)	332.56 ± 14.30	329.237 ± 20.62	0.511
Parafovea Nasal (μm)	344.41 ± 12.09	344.96 ± 15.79	0.682
Choroidal Thickness-temporal (μm)	320.50 ± 88.71	359.96 ± 77.34	0.011
Choroidal Thickness-subfoveal (μm)	329 ± 73.52	384.40 ± 60.56	<0.001
Choroidal Thickness-nasal (μm)	304.23 ± 71.30	359.44 ± 76.04	0.001
TCA	1.294 ± 0.28	1.479 ± 0.25	<0.001
CVI	0.569 ± 0.04	0.566 ± 0.04	0.600
LCA	0.737 ± 0.16	0.837 ± 0.15	0.002
SCP-Whole VD (%)	47.44 ± 2.82	44.09 ± 3.71	<0.001
SCP-Foveal VD (%)	19.59 ± 6.29	17.69 ± 7.90	0.066
SCP-Parafoveal VD (%)	48.33 ± 4.13	43.20 ± 4.82	<0.001
SCP-Perifoveal VD (%)	48.22 ± 2.76	45.09 ± 3.92	<0.001
DCP-Whole VD (%)	46.13 ± 4.90	44.79 ± 4.48	0.131
DCP-Foveal VD (%)	36.32 ± 7.49	36.34 ± 8.54	0.783
DCP-Parafoveal VD (%)	51.44 ± 4.40	51.60 ± 4.42	0.963
DCP-Perifoveal VD (%)	47.35 ± 5.37	45.36 ± 5.05	0.031
FAZ	0.266 ± 0.10	0.252 ± 0.09	0.586
NFA	0.513 ± 0.12	0.566 ± 0.15	0.038
CCPFA	2126.55 ± 101.63	2154.58 ± 152.82	0.010

GCL: ganglion cell layer; IPL: inner plexiform layer; INL: inner nuclear layer; OPL: outer plexiform layer; ONL: outer nuclear layer; RPE: retinal pigment epithelium; IRLs: inner retinal layers; ORLs: outer retinal layers; CMT: central macular thickness; CVI: choroidal vascularity index; TCA: total choroidal area; LCA: luminal choroidal area; SCP: superficial capillary plexus; DCP: deep capillary plexus; VD: vessel density; FAZ: foveal avascular zone; NFA: non-flow area; CCPFA, choriocapillaris flow void area. *p* < 0.05 is statistically significant. * Mann–Whitney U test.

**Table 3:** Selected Spearman correlations between corneal parameters and OCT-derived retinal and choroidal measurements in keratoconus patients.

Corneal Parameter	OCT Parameter	Spearman r	*p* Value	FDR-Adjusted *p*
Astigmatism	INL	0.248	0.027	0.707
Kmaxf	CVI	−0.260	0.028	0.720
TCT	ORL	−0.236	0.035	0.770
SIf	INL	0.230	0.040	0.761
SIf	ORL	0.252	0.024	0.665
SIb	ORL	0.252	0.024	0.665
SIb	Thick-subfoveal	−0.233	0.049	0.793
SIb	TCA	−0.248	0.036	0.761
Total HOA 6 mm	INL	0.260	0.020	0.640
Total HOA 6 mm	ORL	0.230	0.038	0.760
Total Trefoil 6 mm	INL	0.320	0.004	0.500
Total HOA 3 mm	INL	0.290	0.010	0.500
OPD 6 mm	INL	0.300	0.007	0.500
OPD 6 mm	RPE	−0.310	0.006	0.500
OPD 6 mm	CVI	−0.310	0.009	0.500
SR 6 mm	RPE	0.270	0.017	0.580
SR 6 mm	CVI	0.320	0.007	0.500

INL: inner nuclear layer; ONL: outer nuclear layer; ORL: outer retinal layer; RPE: retinal pigment epithelium; CVI: choroidal vascularity index; TCA: total choroidal area; Thick-subfoveal: subfoveal choroidal thickness; HOA: higher-order aberrations; SA: spherical aberration; OPD: optical path difference; SR: Strehl ratio; TCT: thinnest corneal thickness; Kmaxf: maximum keratometry (front surface); SIb: inferior–superior index (back surface); SIf: inferior–superior index (front surface). Spearman rank correlation analysis with false discovery rate correction was applied; *p* < 0.05 and FDR-adjusted *p* < 0.05 were considered statistically significant.

**Table 4:** Selected Spearman correlations between corneal parameters and OCTA-derived vascular metrics in keratoconus patients.

Corneal Parameter	OCTA Parameter	Spearman r	*p* Value	FDR-Adjusted *p*
Astigmatism	SCP—Perifovea	−0.210	0.065	0.830
Kmaxf	DCP—Perifovea	−0.190	0.095	0.830
Kmaxb	SCP—Perifovea	0.200	0.077	0.830
Kmaxb	DCP—Fovea	0.190	0.098	0.830
SIb	FAZ	0.150	0.188	0.890
Total HOA 6 mm	SCP—Whole	−0.007	0.949	0.985
	DCP—Whole	−0.155	0.180	0.882
	FAZ	0.064	0.585	0.905
Total Coma 6 mm	SCP—Whole	−0.046	0.693	0.924
Total HOA 3 mm	SCP—Whole	0.030	0.795	0.951
	DCP—Whole	−0.126	0.280	0.890
	FAZ	0.140	0.229	0.890

SCP: superficial capillary plexus; DCP: deep capillary plexus; FAZ: foveal avascular zone; HOA: higher-order aberrations; OPD: optical path difference; SR: Strehl ratio; Kmaxf: maximum keratometry (front surface); Kmaxb: maximum keratometry (back surface); SIb: inferior–superior index (back surface). Spearman rank correlation analysis with false discovery rate correction was applied; *p* < 0.05 and FDR-adjusted *p* < 0.05 were considered statistically significant.

## Data Availability

The original contributions presented in this study are included in the article/Supplementary Material. Further inquiries can be directed to the corresponding author.

## Supplementary Materials

The following supporting information can be downloaded at: https://www.mdpi.com/article/10.3390/diagnostics16081212/s1, Supplementary Table S1: Complete Spearman correlation dataset between corneal topography parameters and OCT measurements in keratoconus patients. Supplementary Table S2: Spearman correlation analysis between corneal higher-order aberration parameters and OCT-derived retinal and choroidal measurements in keratoconus patients Supplementary Table S3: Comprehensive Spearman correlation analysis between corneal topography parameters and OCT angiography metrics in keratoconus patients. Supplementary Table S4: Spearman correlation analysis between comprehensive corneal higher-order aberration parameters and OCTA parameters in keratoconus patients. Supplementary Table S5: Excel file.

## References

[1] Romero-Jiménez M., Santodomingo-Rubido J., Wolffsohn J.S. (2010). Keratoconus: A review. Contact Lens Anterior Eye.

[2] Santodomingo-Rubido J., Carracedo G., Suzaki A., Villa-Collar C., Vincent S.J., Wolffsohn J.S. (2022). Keratoconus: An updated review. Contact Lens Anterior Eye.

[3] Fard A.M., Patel S.P., Sorkhabi R.D., Salekzamani S., Pezzino E., Nader N.D. (2020). Posterior pole retinal thickness distribution pattern in keratoconus. Int. Ophthalmol..

[4] Moschos M.M., Chatziralli I.P., Koutsandrea C., Siasou G., Droutsas D. (2013). Assessment of the macula in keratoconus: An optical coherence tomography and multifocal electroretinography study. Ophthalmologica.

[5] Leclaire M.D., Storp J.J., Lahme L., Esser E.L., Eter N., Alnawaiseh M. (2024). Reduced Retinal Blood Vessel Densities Measured by Optical Coherence Tomography Angiography in Keratoconus Patients Are Negatively Correlated with Keratoconus Severity. Diagnostics.

[6] Sadeghi J., Barooti Y., Gharaei H., Shoeibi N., Sedaghat M., Yazdani N., Abasi Mehrabadi A., Motamed Shariati M. (2024). Retinal neurovascular assessment and choroidal vascularity index in patients with keratoconus. Sci. Rep..

[7] Erdinest N., London N., Landau D., Barbara R., Barbara A., Naroo S.A. (2024). Higher order aberrations in keratoconus. Int. Ophthalmol..

[8] Pierro L., Bianco L., Bertuzzi F., Arrigo A., Saladino A., Distefano A., Berni A., Knutsson K.A., Rama P., Bandello F. (2023). New Findings in Early-Stage Keratoconus: Lamina Cribrosa Curvature, Retinal Nerve Fiber Layer Thickness, and Vascular Perfusion. Am. J. Ophthalmol..

[9] Hashemi H., Heirani M., Ambrósio R., Hafezi F., Naroo S.A., Khorrami-Nejad M. (2022). The link between Keratoconus and posterior segment parameters: An updated, comprehensive review. Ocul. Surf..

[10] Bayat K., Pooyan P., Feizi S., Ahmadieh H., Hafezi F., Pourhoseingholi M.A., Fekri S., Sarraf D. (2026). Structural alterations in the retina and choroid of keratoconus patients detected by optical coherence tomography: A systematic review and meta-analysis. Surv. Ophthalmol..

[11] Gordon-Shaag A., Millodot M., Ifrah R., Shneor E. (2012). Aberrations and topography in normal, keratoconus-suspect, and keratoconic eyes. Optom. Vis. Sci..

[12] Xu Z., Li W., Jiang J., Zhuang X., Chen W., Peng M., Wang J., Lu F., Shen M., Wang Y. (2017). Characteristic of entire corneal topography and tomography for the detection of sub-clinical keratoconus with Zernike polynomials using Pentacam. Sci. Rep..

[13] Vidal-Oliver L., Gallego-Pinazo R., Dolz-Marco R. (2024). Astigmatism Influences Quantitative and Qualitative Analysis in Optical Coherence Tomography Angiography Imaging. Transl. Vis. Sci. Technol..

[14] Anvari P., Ashrafkhorasani M., Habibi A., Falavarjani K.G. (2021). Artifacts in Optical Coherence Tomography Angiography. J. Ophthalmic Vis. Res..

[15] Sahebjada S., Amirul Islam F.M., Wickremasinghe S., Daniell M., Baird P.N. (2015). Assessment of macular parameter changes in patients with keratoconus using optical coherence tomography. J. Ophthalmol..

[16] Uzunel U.D., Küsbeci T., Yüksel B. (2017). Does the Stage of Keratoconus Affect Optical Coherence Tomography Measurements?. Semin. Ophthalmol..

[17] Wylęgała A., Szkodny D., Fiolka R., Wylęgała E. (2022). Assessment of the Retinal Vessels in Keratoconus: An OCT Angiography Study. J. Clin. Med..

[18] Aydemir G.A., Kocabaş D.O., Bilen A., Aydemir E., Bayat A.H., Oren B., Kiziltoprak H. (2023). Evaluation of Retinal Layer Thicknesses in Patients with Keratoconus Using Retinal Layer Segmentation Analysis. Klin. Monatsblatter Augenheilkd..

[19] Özsaygılı C., Yıldırım Y. (2021). The Relationship Between Keratoconus Stage and the Thickness of the Retinal Layers. Turk. J. Ophthalmol..

[20] Gutierrez-Bonet R., Ruiz-Medrano J., Peña-Garcia P., Catanese M., Sadeghi Y., Hashemi K., Gabison E., Ruiz-Moreno J.M. (2018). Macular Choroidal Thickening in Keratoconus Patients: Swept-Source Optical Coherence Tomography Study. Transl. Vis. Sci. Technol..

[21] Pinheiro-Costa J., Viana Pinto J., Perestrelo S., Beato J.N., Torrão L., Brandão E., Carneiro Â., Madeira M.D., Falcão-Reis F. (2019). Increased Choroidal Thickness in Keratoconus Patients: Perspectives in the Disease Pathophysiology. J. Ophthalmol..

[22] Akkaya S. (2018). Macular and Peripapillary Choroidal Thickness in Patients with Keratoconus. Ophthalmic Surg. Lasers Imaging Retin..

[23] Dogan B., Bozdogan Y.C., Gedik B., Erol M.K., Bulut M., Duman F. (2023). Optic disc and retinal vessel densities assessment by optical coherence tomography angiography in patients with keratoconus. Photodiagn. Photodyn. Ther..

[24] Zırtıloğlu S., Alikma M.S., Akarsu Acar O.P., Furuncuoglu U., Guven F. (2023). Evaluation of the optic nerve head and macular vessel density in keratoconus patients using optical coherence tomography angiography- a cross-sectional study. Eur. J. Ophthalmol..

