# Supplementary material for: Corneal Higher-Order Aberrations and Posterior Segment Changes in Keratoconus: A Multimodal OCT and OCTA Study
Source: Diagnostics (Basel). 2026 Apr 18;16(8):1212. doi: 10.3390/diagnostics16081212 (PMC13115241; doi:10.3390/diagnostics16081212)
Supplement: Supplementary file 1 [file diagnostics-16-01212-s001.zip › Supplementary Table S1.pdf]

Supplementary Table S1. Complete Spearman correlation dataset between corneal topography parameters and OCT measurements in keratoconus patients

Topography	OCT parameter	Spearman r	p value	FDR-adjusted
**Astigmatism**	GCL	-0,017	0,881	0,968046
**Astigmatism**	IPL	-0,054	0,634	0,905263
**Astigmatism**	INL	**0,248**	**0,027**	0,706909
**Astigmatism**	OPL	0,046	0,683	0,918319
**Astigmatism**	ONL	-0,218	0,052	0,813913
**Astigmatism**	RPE	-0,153	0,176	0,88
**Astigmatism**	IRL	0,021	0,853	0,904537
**Astigmatism**	ORL	0,06	0,596	0,887510
**Astigmatism**	CMT	0,065	0,56	0,887510
**Astigmatism**	Thick-subfov	0,103	0,391	0,904537
**Astigmatism**	TCA	0,052	0,664	0,914003
**Astigmatism**	CVI	-0,193	0,104	0,830204
**Astigmatism**	LCA	-0,067	0,577	0,904537
**Kmaxf**	GCL	-0,104	0,36	0,88751
**Kmaxf**	IPL	-0,074	0,512	0,894334
**Kmaxf**	INL	0,189	0,093	0,834462
**Kmaxf**	OPL	-0,056	0,62	0,905263
**Kmaxf**	ONL	0,04	0,724	0,931689
**Kmaxf**	RPE	-0,173	0,124	0,854328
**Kmaxf**	IRL	0,039	0,73	0,9344
**Kmaxf**	ORL	0,169	0,134	0,854328
**Kmaxf**	CMT	0,104	0,357	0,88751
**Kmaxf**	Thick-subfov	0,045	0,709	0,928145
**Kmaxf**	TCA	0,024	0,84	0,957369
**Kmaxf**	CVI	**-0,26**	**0,028**	0,72
**Kmaxf**	LCA	-0,072	0,546	0,902662
**Kmaxb**	GCL	0,101	0,374	0,88751
**Kmaxb**	IPL	0,067	0,554	0,904538
**Kmaxb**	INL	-0,173	0,125	0,854328
**Kmaxb**	OPL	0,7	0,535	0,902662
**Kmaxb**	ONL	0,018	0,877	0,966243
**Kmaxb**	RPE	-0,011	0,921	0,978865
**Kmaxb**	IRL	0,02	0,861	0,960372
**Kmaxb**	ORL	-0,213	0,057	0,834462
**Kmaxb**	CMT	-0,05	0,661	0,912598
**Kmaxb**	Thick-subfov	0,077	0,519	0,897191
**Kmaxb**	TCA	0,043	0,718	0,931689
**Kmaxb**	CVI	0,169	0,157	0,854328
**Kmaxb**	LCA	0,084	0,483	0,892518

**TCT**	GCL	0,069	0,542	0,902662
**TCT**	IPL	-0,009	0,934	0,978865
**TCT**	INL	-0,063	0,578	0,904538
**TCT**	OPL	0,059	0,604	0,905263
**TCT**	ONL	0,076	0,502	0,892518
**TCT**	RPE	-0,106	0,351	0,88751
**TCT**	IRL	0,038	0,739	0,938413
**TCT**	ORL	**-0,236**	**0,035**	0,770233
**TCT**	CMT	-0,01	0,931	0,978865
**TCT**	Thick-subfov	-0,186	0,118	0,844138
**TCT**	TCA	-0,146	0,222	0,88751
**TCT**	CVI	0,031	0,795	0,951344
**TCT**	LCA	-0,126	0,29	0,88751
**SIf**	GCL	-0,041	0,721	0,93168
**SIf**	IPL	-0,034	0,762	0,943918
**SIf**	INL	**0,23**	**0,040**	0,761379
**SIf**	OPL	-0,01	0,931	0,978865
**SIf**	ONL	-0,134	0,236	0,88751
**SIf**	RPE	-0,019	0,87	0,962952
**SIf**	IRL	0,032	0,779	0,949839
**SIf**	ORL	**0,252**	**0,024**	0,664615
**SIf**	CMT	0,104	0,359	0,88751
**SIf**	Thick-subfov	-0,123	0,302	0,88751
**Sif**	TCA	-0,197	0,097	0,830204
**SIf**	CVI	-0,154	0,196	0,88751
**SIf**	LCA	-0,217	0,067	0,83
**SIb**	GCL	-0,095	0,402	0,88751
**SIb**	IPL	-0,094	0,408	0,88751
**SIb**	INL	0,183	0,104	0,830204
**SIb**	OPL	-0,069	0,545	0,902662
**SIb**	ONL	-0,177	0,116	0,8352
**SIb**	RPE	0,117	0,302	0,88751
**SIb**	IRL	-0,065	0,567	0,904538
**SIb**	ORL	**0,252**	**0,024**	0,664615
**SIb**	CMT	0,004	0,971	0,989554
**SIb**	Thick-subfov	**-0,233**	**0,049**	0,792809
**SIb**	TCA	**-0,248**	**0,036**	0,761379
**SIb**	CVI	-0,067	0,576	0,904538
**SIb**	LCA	-0,221	0,062	0,83

Kmaxf: maximum keratometry front; Kmaxb: maximum keratometry back; TCT:thinnest corneal thickness; SIf: surface irregularity index (front); SIb: surface irregularity index (back); GCL: ganglion cell layer; IPL: inner plexiform layer; INL: inner nuclear layer; OPL: outer plexiform layer; ONL: outer nuclear layer; RPE: retinal pigment epithelium; IRL: inner retinal layers; ORL: outer retinal layers; CMT: central macular thickness; Thick-subfov: subfoveal choroidal thickness; TCA: total choroidal area; CVI: choroidal vascularity index; LCA: luminal choroidal area.

P<0.05 is statistically significant. FDR <0.05 is statistically significant.
