# Supplementary material for: Corneal Higher-Order Aberrations and Posterior Segment Changes in Keratoconus: A Multimodal OCT and OCTA Study
Source: Diagnostics (Basel). 2026 Apr 18;16(8):1212. doi: 10.3390/diagnostics16081212 (PMC13115241; doi:10.3390/diagnostics16081212)
Supplement: Supplementary file 1 [file diagnostics-16-01212-s001.zip › Supplementary Table S2.pdf]

Supplementary Table S2. Spearman correlation analysis between corneal higher-order aberration parameters and OCT-derived retinal and choroidal measurements in keratoconus patients

Aberration parameter	OCT parameter	Spearman r	p value	FDR-adjusted
**Total HOA 6mm**	GCL	0,006	0,961	0,987633262
**Total HOA 6mm**	IPL	-0,061	0,591	0,904537815
**Total HOA 6mm**	INL	**0,26**	**0,02**	0.64
**Total HOA 6mm**	OPL	0,03	0,79	0,951344262
**Total HOA 6mm**	ONL	-0,112	0,322	0,889931787
**Total HOA 6mm**	RPE	-0,196	0,082	0,83
**Total HOA 6mm**	IRL	0,071	0,531	0,901698113
**Total HOA 6mm**	ORL	**0,232**	**0,038**	0,76137931
**Total HOA 6mm**	CMT	0,154	0,173	0,871048951
**Total HOA 6mm**	Thick-subfov	-0,014	0,907	0,978864629
**Total HOA 6mm**	TCA	-0,06	0,614	0,905263158
**Total HOA 6mm**	CVI	-0,216	0,069	0,83
**Total HOA 6mm**	LCA	-0,131	0,271	0,889931787
**Total Coma 6mm**	GCL	-0,054	0,636	0,905263158
**Total Coma 6mm**	IPL	-0,126	0,264	0,889931787
**Total Coma 6mm**	INL	0,165	0,143	0,851152416
**Total Coma 6mm**	OPL	-0,001	0,99	0,995530726
**Total Coma 6mm**	ONL	-0,143	0,207	0,887510204
**Total Coma 6mm**	RPE	-0,201	0,074	0.83
**Total Coma 6mm**	IRL	0,001	0,995	0,997077244
**Total Coma 6mm**	ORL	0,186	0,098	0,830204082
**Total Coma 6mm**	CMT	0,076	0,501	0,892518337
**Total Coma 6mm**	Thick-subfov	-0,006	0,96	0,987633262
**Total Coma 6mm**	TCA	-0,109	0,362	0,889931787
**Total Coma 6mm**	CVI	-0,21	0,077	0,83
**Total Coma 6mm**	LCA	-0,176	0,14	0,851152416
**Total SA 6mm**	GCL	0,061	0,591	0,904537815
**Total SA 6mm**	IPL	0,033	0,774	0,949369676
**Total SA 6mm**	INL	**0,232**	**0,038**	0,76137931
**Total SA 6mm**	OPL	0,074	0,516	0,895228916
**Total SA 6mm**	ONL	0,153	0,176	0,876955017
**Total SA 6mm**	RPE	-0,18	0,109	0,830204082
**Total SA 6mm**	IRL	0,173	0,124	0,850604651
**Total SA 6mm**	ORL	0,051	0,653	0,909400387
**Total SA 6mm**	CMT	0,169	0,134	0,851152416
**Total SA 6mm**	Thick-subfov	0,08	0,506	0,892518337
**Total SA 6mm**	TCA	0,115	0,337	0,889931787
**Total SA 6mm**	CVI	-0,069	0,567	0,904537815
**Total SA 6mm**	LCA	0,091	0,446	0,889931787

**Total Trefoil 6mm**	GCL	0,069	0,541	0,902662116
**Total Trefoil 6mm**	IPL	-0,025	0,823	0,956521739
**Total Trefoil 6mm**	INL	**0,315**	**0,004**	0,496551724
**Total Trefoil 6mm**	OPL	0,074	0,517	0,895884477
**Total Trefoil 6mm**	ONL	-0,116	0,305	0,889931787
**Total Trefoil 6mm**	RPE	-0,101	0,373	0,889931787
**Total Trefoil 6mm**	IRL	0,088	0,44	0,889931787
**Total Trefoil 6mm**	ORL	0,057	0,615	0,905263158
**Total Trefoil 6mm**	CMT	0,138	0,221	0,889931787
**Total Trefoil 6mm**	Thick-subfov	-0,087	0,467	0,892518337
**Total Trefoil 6mm**	TCA	-0,1	0,401	0,889931787
**Total Trefoil 6mm**	CVI	-0,095	0,426	0,889931787
**Total Trefoil 6mm**	LCA	-0,108	0,365	0,889931787
**Total HOA 3mm**	GCL	0,006	0,955	0,987221823
**Total HOA 3mm**	IPL	-0,055	0,627	0,905263158
**Total HOA 3mm**	INL	**0,287**	**0,01**	0,496551724
**Total HOA 3mm**	OPL	0,011	0,926	0,978864629
**Total HOA 3mm**	ONL	-0,161	0,153	0,851152416
**Total HOA 3mm**	RPE	-0,181	0,108	0,830204082
**Total HOA 3mm**	IRL	0,045	0,689	0,923798883
**Total HOA 3mm**	ORL	0,179	0,112	0,830204082
**Total HOA 3mm**	CMT	0,12	0,288	0,889931787
**Total HOA 3mm**	Thick-subfov	-0,108	0,364	0,889931787
**Total HOA 3mm**	TCA	-0,113	0,344	0,889931787
**Total HOA 3mm**	CVI	-0,172	0,148	0,851152416
**Total HOA 3mm**	LCA	-0,17	0,154	0,851152416
**Total Coma 3mm**	GCL	-0,04	0,727	0,933880464
**Total Coma 3mm**	IPL	-0,061	0,592	0,904537815
**Total Coma 3mm**	INL	**0,229**	**0,041**	0,76137931
**Total Coma 3mm**	OPL	-0,03	0,789	0,951344262
**Total Coma 3mm**	ONL	-0,21	0,061	0,83
**Total Coma 3mm**	RPE	-0,193	0,086	0,830204082
**Total Coma 3mm**	IRL	0,01	0,933	0,978864629
**Total Coma 3mm**	ORL	0,203	0,071	0,83
**Total Coma 3mm**	CMT	0,086	0,449	0,889931787
**Total Coma 3mm**	Thick-subfov	-0,056	0,638	0,905263158
**Total Coma 3mm**	TCA	-0,106	0,374	0,889931787
**Total Coma 3mm**	CVI	-0,179	0,133	0,851152416
**Total Coma 3mm**	LCA	-0,169	0,157	0,851152416
**Total SA 3mm**	GCL	-0,191	0,091	0,830204082
**Total SA 3mm**	IPL	-0,139	0,22	0,889931787
**Total SA 3mm**	INL	0,092	0,418	0,887510204
**Total SA 3mm**	OPL	-0,086	0,446	0,887510204
**Total SA 3mm**	ONL	-0,159	0,159	0,851152416
**Total SA 3mm**	RPE	**-0,249**	**0,026**	0,706415094
**Total SA 3mm**	IRL	-0,13	0,252	0,887510204
**Total SA 3mm**	ORL	0,122	0,282	0,887510204
**Total SA 3mm**	CMT	-0,098	0,387	0,887510204

**Total SA 3mm**	Thick-subfov	0,189	0,111	0,830204082
**Total SA 3mm**	TCA	0,186	0,119	0,84
**Total SA 3mm**	CVI	-0,084	0,485	0,892518337
**Total SA 3mm**	LCA	0,109	0,36	0,887510204
**Total Trefoil 3mm**	GCL	0,043	0,704	0,926654479
**Total Trefoil 3mm**	IPL	-0,029	0,795	0,951344262
**Total Trefoil 3mm**	INL	**0,272**	**0,015**	0,582857143
**Total Trefoil 3mm**	OPL	0,024	0,835	0,957368833
**Total Trefoil 3mm**	ONL	-0,14	0,216	0,889931787
**Total Trefoil 3mm**	RPE	-0,049	0,668	0,914003795
**Total Trefoil 3mm**	IRL	0,052	0,644	0,905263158
**Total Trefoil 3mm**	ORL	0,109	0,336	0,887510204
**Total Trefoil 3mm**	CMT	0,106	0,35	0,887510204
**Total Trefoil 3mm**	Thick-subfov	-0,222	0,061	0,83
**Total Trefoil 3mm**	TCA	-0,206	0,083	0,83
**Total Trefoil 3mm**	CVI	-0,051	0,67	0,91450237
**Total Trefoil 3mm**	LCA	-0,208	0,08	0,83
**Ant HOA 6mm**	GCL	-0,04	0,724	0,931689008
**Ant HOA 6mm**	IPL	-0,085	0,451	0,887510204
**Ant HOA 6mm**	INL	**0,227**	**0,043**	0,76137931
**Ant HOA 6mm**	OPL	-0,008	0,943	0,98186551
**Ant HOA 6mm**	ONL	-0,137	0,226	0,887510204
**Ant HOA 6mm**	RPE	-0,14	0,214	0,889931787
**Ant HOA 6mm**	IRL	0,024	0,833	0,957368833
**Ant HOA 6mm**	ORL	**0,238**	**0,033**	0,76137931
**Ant HOA 6mm**	CMT	0,105	0,353	0,887510204
**Ant HOA 6mm**	Thick-subfov	-0,042	0,723	0,931689008
**Ant HOA 6mm**	TCA	-0,079	0,507	0,892518337
**Ant HOA 6mm**	CVI	-0,195	0,1	0,830204082
**Ant HOA 6mm**	LCA	-0,139	0,245	0,887510204
**Ant HOA 3mm**	GCL	-0,046	0,683	0,918319328
**Ant HOA 3mm**	IPL	-0,079	0,485	0,892518337
**Ant HOA 3mm**	INL	0,211	0,06	0,83
**Ant HOA 3mm**	OPL	-0,034	0,763	0,943917526
**Ant HOA 3mm**	ONL	**-0,223**	**0,047**	0,76137931
**Ant HOA 3mm**	RPE	-0,085	0,453	0,887510204
**Ant HOA 3mm**	IRL	-0,023	0,842	0,957368833
**Ant HOA 3mm**	ORL	**0,228**	**0,042**	0,76137931
**Ant HOA 3mm**	CMT	0,053	0,639	0,905263158
**Ant HOA 3mm**	Thick-subfov	-0,131	0,274	0,887510204
**Ant HOA 3mm**	TCA	-0,133	0,264	0,887510204
**Ant HOA 3mm**	CVI	-0,144	0,229	0,887510204
**Ant HOA 3mm**	LCA	-0,172	0,148	0,851152416
**OPD 6**	GCL	0,038	0,735	0,937466785
**OPD 6**	IPL	-0,021	0,854	0,95962675
**OPD 6**	INL	**0,299**	**0,007**	0,496551724
**OPD 6**	OPL	0,046	0,683	0,918319328
**OPD 6**	ONL	-0,104	0,36	0,887510204
**OPD 6**	RPE	**-0,306**	**0,006**	0,496551724

**OPD 6**	IRL	0,108	0,343	0,887510204
**OPD 6**	ORL	0,092	0,415	0,887510204
**OPD 6**	CMT	0,172	0,127	0,850604651
**OPD 6**	Thick-subfov	0,07	0,557	0,904537815
**OPD 6**	TCA	0,055	0,646	0,905783836
**OPD 6**	CVI	**-0,305**	**0,009**	0,496551724
**OPD 6**	LCA	-0,092	0,44	0,887510204
**SR6**	GCL	0,067	0,558	0,904537815
**SR6**	IPL	0,107	0,347	0,887510204
**SR6**	INL	-0,185	0,101	0,830204082
**SR6**	OPL	0,097	0,391	0,887510204
**SR6**	ONL	0,089	0,433	0,887510204
**SR6**	RPE	**0,266**	**0,017**	0,582857143
**SR6**	IRL	0,005	0,965	0,987633262
**SR6**	ORL	-0,049	0,668	0,914003795
**SR6**	CMT	-0,059	0,605	0,905263158
**SR6**	Thick-subfov	-0,059	0,621	0,905263158
**SR6**	TCA	-0,073	0,541	0,902662116
**SR6**	CVI	**0,317**	**0,007**	0,496551724
**SR6**	LCA	0,095	0,428	0,887510204

RMS: root mean square; HOA: higher-order aberrations; SA: spherical aberration; OPD: optical path difference; SR: Strehl ratio; GCL: ganglion cell layer; IPL: inner plexiform layer; INL: inner nuclear layer; OPL: outer plexiform layer; ONL: outer nuclear layer; RPE: retinal pigment epithelium; IRL: inner retinal layers; ORL: outer retinal layers; CMT: central macular thickness; Thick-subfov: subfoveal choroidal thickness; TCA: total choroidal area; CVI: choroidal vascularity index; LCA: luminal choroidal area.

P<0.05 is statistically significant. FDR <0.05 is statistically significant.
