# Supplementary material for: Corneal Higher-Order Aberrations and Posterior Segment Changes in Keratoconus: A Multimodal OCT and OCTA Study
Source: Diagnostics (Basel). 2026 Apr 18;16(8):1212. doi: 10.3390/diagnostics16081212 (PMC13115241; doi:10.3390/diagnostics16081212)
Supplement: Supplementary file 1 [file diagnostics-16-01212-s001.zip › Supplementary Table S3.pdf]

Supplementary Table S3. Comprehensive Spearman correlation analysis between corneal topography parameters and OCT angiography metrics in keratoconus patients.

Topography	OCTA parameter	Spearman r	p value	FDR-adjusted
**Astigmatism**	SCP-Whole	-0,166	0,151	0,887510204
**Astigmatism**	SCP-fovea	-0,029	0,804	0,951344262
**Astigmatism**	SCP- parafovea	-0,114	0,329	0,887510204
**Astigmatism**	SCP-perifovea	-0,213	0,065	0,83
**Astigmatism**	DCP-Whole	-0,07	0,551	0,902662116
**Astigmatism**	DCP-fovea	-0,86	0,462	0,89088
**Astigmatism**	DCP-parafovea	-0,74	0,527	0,89808284
**Astigmatism**	DCP-perifovea	-0,078	0,506	0,892518337
**Astigmatism**	FAZ	0,148	0,201	0,887510204
**Kmaxf**	SCP-Whole	-0,093	0,423	0,887510204
**Kmaxf**	SCP-fovea	-0,055	0,635	0,905263158
**Kmaxf**	SCP- parafovea	-0,065	0,579	0,904537815
**Kmaxf**	SCP-perifovea	-0,179	0,123	0,850604651
**Kmaxf**	DCP-Whole	-0,178	0,123	0,850604651
**Kmaxf**	DCP-fovea	-0,158	0,173	0,871048951
**Kmaxf**	DCP-parafovea	-0,063	0,588	0,904537815
**Kmaxf**	DCP-perifovea	-0,193	0,095	0,830204082
**Kmaxf**	FAZ	0,126	0,279	0,887510204
**Kmaxb**	SCP-Whole	0,103	0,374	0,887510204
**Kmaxb**	SCP-fovea	0,066	0,572	0,904537815
**Kmaxb**	SCP- parafovea	0,064	0,582	0,904537815
**Kmaxb**	SCP-perifovea	0,204	0,077	0,83
**Kmaxb**	DCP-Whole	0,132	0,257	0,887510204
**Kmaxb**	DCP-fovea	0,191	0,098	0,830204082
**Kmaxb**	DCP-parafovea	0,045	0,698	0,924673413
**Kmaxb**	DCP-perifovea	0,176	0,127	0,850604651
**Kmaxb**	FAZ	-0,187	0,106	0,830204082
**TCT**	SCP-Whole	0,021	0,855	0,95962675
**TCT**	SCP-fovea	-0,123	0,29	0,887510204
**TCT**	SCP- parafovea	0,009	0,942	0,98186551
**TCT**	SCP-perifovea	0,07	0,548	0,902662116
**TCT**	DCP-Whole	-0,007	0,954	0,986896552
**TCT**	DCP-fovea	-0,008	0,944	0,982196532
**TCT**	DCP-parafovea	-0,062	0,598	0,904537815
**TCT**	DCP-perifovea	0,005	0,976	0,991142454
**TCT**	FAZ	-0,028	0,809	0,952542927

**SIf**	SCP-Whole	0,036	0,761	0,943917526
**SIf**	SCP-fovea	0,109	0,349	0,887510204
**SIf**	SCP- parafovea	-0,016	0,893	0,974181818
**SIf**	SCP-perifovea	-0,038	0,743	0,939130435
**SIf**	DCP-Whole	-0,087	0,453	0,887510204
**SIf**	DCP-fovea	-0,095	0,416	0,887510204
**SIf**	DCP-parafovea	-0,021	0,856	0,95962675
**SIf**	DCP-perifovea	-0,167	0,15	0,851152416
**SIf**	FAZ	0,059	0,614	0,905263158
**SIb**	SCP-Whole	-0,009	0,939	0,980536621
**SIb**	SCP-fovea	0,041	0,725	0,932142857
**SIb**	SCP- parafovea	-0,091	0,435	0,887510204
**SIb**	SCP-perifovea	-0,108	0,351	0,887510204
**SIb**	DCP-Whole	-0,03	0,797	0,951344262
**SIb**	DCP-fovea	-0,148	0,201	0,887510204
**SIb**	DCP-parafovea	0,038	0,745	0,939130435
**SIb**	DCP-perifovea	-0,125	0,28	0,887510204
**SIb**	FAZ	0,153	0,188	0,887510204

Kmaxf: maximum keratometry front; Kmaxb: maximum keratometry back; TCT:thinnest corneal thickness; SIf: surface irregularity index (front); SIb: surface irregularity index (back); SCP: superficial capillary plexus; DCP: deep capillary plexus; FAZ: foveal avascular zone.

P<0.05 is statistically significant. FDR <0.05 is statistically significant.
