# Supplementary material for: Corneal Higher-Order Aberrations and Posterior Segment Changes in Keratoconus: A Multimodal OCT and OCTA Study
Source: Diagnostics (Basel). 2026 Apr 18;16(8):1212. doi: 10.3390/diagnostics16081212 (PMC13115241; doi:10.3390/diagnostics16081212)
Supplement: Supplementary file 1 [file diagnostics-16-01212-s001.zip › Supplementary Table S4.pdf]

**Supplementary Table S4.** Spearman correlation analysis between comprehensive corneal higher-order aberration parameters and OCTA parameters in keratoconus patients.

| Aberration parameter | OCTA parameter | Spearman r | p value | FDR-adjusted |
|---|---|---|---|---|
| **Total HOA 6mm** | SCP-Whole | -0,007 | 0,949 | 0,984553314 |
| **Total HOA 6mm** | SCP-fovea | 0,093 | 0,424 | 0,889931787 |
| **Total HOA 6mm** | SCP- parafovea | -0,057 | 0,627 | 0,905263158 |
| **Total HOA 6mm** | SCP-perifovea | -0,067 | 0,565 | 0,904537815 |
| **Total HOA 6mm** | DCP-Whole | -0,155 | 0,18 | 0,881632653 |
| **Total HOA 6mm** | DCP-fovea | -0,015 | 0,897 | 0,976327 |
| **Total HOA 6mm** | DCP-parafovea | -0,15 | 0,197 | 0,889931787 |
| **Total HOA 6mm** | DCP-perifovea | -0,182 | 0,115 | 0,8352 |
| **Total HOA 6mm** | FAZ | 0,064 | 0,585 | 0,904537815 |
| **Total Coma 6mm** | SCP-Whole | -0,046 | 0,693 | 0,924477336 |
| **Total Coma 6mm** | SCP-fovea | 0,072 | 0,538 | 0,902662116 |
| **Total Coma 6mm** | SCP- parafovea | -0,089 | 0,446 | 0,889931787 |
| **Total Coma 6mm** | SCP-perifovea | -0,115 | 0,321 | 0,889931787 |
| **Total Coma 6mm** | DCP-Whole | -0,202 | 0,08 | 0,83 |
| **Total Coma 6mm** | DCP-fovea | -0,064 | 0,581 | 0,904537815 |
| **Total Coma 6mm** | DCP-parafovea | -0,133 | 0,254 | 0,889931787 |
| **Total Coma 6mm** | DCP-perifovea | -0,224 | 0,052 | 0,80516129 |
| **Total Coma 6mm** | FAZ | 0,086 | 0,461 | 0,89088 |
| **Total SA 6mm** | SCP-Whole | 0,052 | 0,657 | 0,910567854 |
| **Total SA 6mm** | SCP-fovea | 0,077 | 0,507 | 0,892518337 |
| **Total SA 6mm** | SCP- parafovea | 0,046 | 0,692 | 0,924477336 |
| **Total SA 6mm** | SCP-perifovea | 0,042 | 0,719 | 0,931689008 |
| **Total SA 6mm** | DCP-Whole | 0,071 | 0,543 | 0,902662116 |
| **Total SA 6mm** | DCP-fovea | 0,117 | 0,313 | 0,889931787 |
| **Total SA 6mm** | DCP-parafovea | -0,055 | 0,638 | 0,905263158 |
| **Total SA 6mm** | DCP-perifovea | 0,065 | 0,576 | 0,904537815 |
| **Total SA 6mm** | FAZ | -0,045 | 0,701 | 0,926091743 |
| **Total Trefoil 6mm** | SCP-Whole | 0,034 | 0,767 | 0,945616438 |
| **Total Trefoil 6mm** | SCP-fovea | 0,091 | 0,437 | 0,889931787 |
| **Total Trefoil 6mm** | SCP- parafovea | 0,027 | 0,818 | 0,956103896 |
| **Total Trefoil 6mm** | SCP-perifovea | -0,014 | 0,905 | 0,978864629 |
| **Total Trefoil 6mm** | DCP-Whole | -0,046 | 0,694 | 0,924477336 |
| **Total Trefoil 6mm** | DCP-fovea | 0,057 | 0,623 | 0,905263158 |
| **Total Trefoil 6mm** | DCP-parafovea | -0,142 | 0,222 | 0,889931787 |
| **Total Trefoil 6mm** | DCP-perifovea | -0,077 | 0,511 | 0,89433414 |
| **Total Trefoil 6mm** | FAZ | 0,096 | 0,41 | 0,889931787 |
| **Total HOA 3mm** | SCP-Whole | 0,03 | 0,795 | 0,951344262 |
| **Total HOA 3mm** | SCP-fovea | 0,094 | 0,42 | 0,889931787 |
| **Total HOA 3mm** | SCP- parafovea | 0,027 | 0,815 | 0,954146341 |
| **Total HOA 3mm** | SCP-perifovea | 0,024 | 0,837 | 0,957368833 |
| **Total HOA 3mm** | DCP-Whole | -0,126 | 0,28 | 0,889931787 |
| **Total HOA 3mm** | DCP-fovea | -0,032 | 0,785 | 0,951344262 |
| **Total HOA 3mm** | DCP-parafovea | -0,15 | 0,195 | 0,889931787 |
| **Total HOA 3mm** | DCP-perifovea | -0,169 | 0,145 | 0,851152416 |

| | | | | |
|---|---|---|---|---|
| **Total HOA 3mm** | FAZ | 0,14 | 0,229 | 0,889931787 |
| **Total Coma 3mm** | SCP-Whole | 0,019 | 0,868 | 0,962951576 |
| **Total Coma 3mm** | SCP-fovea | 0,093 | 0,422 | 0,889931787 |
| **Total Coma 3mm** | SCP- parafovea | -0,023 | 0,846 | 0,957368833 |
| **Total Coma 3mm** | SCP-perifovea | -0,043 | 0,715 | 0,931382114 |
| **Total Coma 3mm** | DCP-Whole | -0,105 | 0,365 | 0,889931787 |
| **Total Coma 3mm** | DCP-fovea | -0,055 | 0,635 | 0,905263158 |
| **Total Coma 3mm** | DCP-parafovea | -0,102 | 0,38 | 0,889931787 |
| **Total Coma 3mm** | DCP-perifovea | -0,149 | 0,199 | 0,889931787 |
| **Total Coma 3mm** | FAZ | 0,125 | 0,283 | 0,889931787 |
| **Total SA 3mm** | SCP-Whole | 0,092 | 0,428 | 0,889931787 |
| **Total SA 3mm** | SCP-fovea | -0,033 | 0,779 | 0,949839119 |
| **Total SA 3mm** | SCP- parafovea | 0,102 | 0,379 | 0,889931787 |
| **Total SA 3mm** | SCP-perifovea | 0,099 | 0,395 | 0,889931787 |
| **Total SA 3mm** | DCP-Whole | -0,088 | 0,447 | 0,889931787 |
| **Total SA 3mm** | DCP-fovea | -0,142 | 0,222 | 0,889931787 |
| **Total SA 3mm** | DCP-parafovea | -0,017 | 0,884 | 0,96950495 |
| **Total SA 3mm** | DCP-perifovea | -0,063 | 0,59 | 0,904537815 |
| **Total SA 3mm** | FAZ | 0,182 | 0,116 | 0,8352 |
| **Total Trefoil 3mm** | SCP-Whole | 0,073 | 0,532 | 0,902332155 |
| **Total Trefoil 3mm** | SCP-fovea | 0,062 | 0,596 | 0,904537815 |
| **Total Trefoil 3mm** | SCP- parafovea | 0,061 | 0,602 | 0,905263158 |
| **Total Trefoil 3mm** | SCP-perifovea | 0,032 | 0,784 | 0,951344262 |
| **Total Trefoil 3mm** | DCP-Whole | -0,068 | 0,56 | 0,904537815 |
| **Total Trefoil 3mm** | DCP-fovea | -0,001 | 0,995 | 0,997077244 |
| **Total Trefoil 3mm** | DCP-parafovea | -0,139 | 0,231 | 0,887510204 |
| **Total Trefoil 3mm** | DCP-perifovea | -0,096 | 0,407 | 0,887510204 |
| **Total Trefoil 3mm** | FAZ | 0,116 | 0,317 | 0,887510204 |
| **Ant HOA 6mm** | SCP-Whole | -0,024 | 0,84 | 0,957368833 |
| **Ant HOA 6mm** | SCP-fovea | 0,055 | 0,636 | 0,905263158 |
| **Ant HOA 6mm** | SCP- parafovea | -0,09 | 0,441 | 0,887510204 |
| **Ant HOA 6mm** | SCP-perifovea | -0,091 | 0,435 | 0,887510204 |
| **Ant HOA 6mm** | DCP-Whole | -0,128 | 0,272 | 0,887510204 |
| **Ant HOA 6mm** | DCP-fovea | -0,061 | 0,6 | 0,905263158 |
| **Ant HOA 6mm** | DCP-parafovea | -0,097 | 0,405 | 0,887510204 |
| **Ant HOA 6mm** | DCP-perifovea | -0,158 | 0,172 | 0,871048951 |
| **Ant HOA 6mm** | FAZ | 0,104 | 0,372 | 0,887510204 |
| **Ant HOA 3mm** | SCP-Whole | 0,008 | 0,943 | 0,98186551 |
| **Ant HOA 3mm** | SCP-fovea | 0,055 | 0,638 | 0,905263158 |
| **Ant HOA 3mm** | SCP- parafovea | -0,089 | 0,447 | 0,887510204 |
| **Ant HOA 3mm** | SCP-perifovea | -0,049 | 0,674 | 0,915622642 |
| **Ant HOA 3mm** | DCP-Whole | -0,095 | 0,415 | 0,887510204 |
| **Ant HOA 3mm** | DCP-fovea | -0,103 | 0,374 | 0,887510204 |
| **Ant HOA 3mm** | DCP-parafovea | -0,065 | 0,579 | 0,904537815 |
| **Ant HOA 3mm** | DCP-perifovea | -0,143 | 0,217 | 0,889931787 |
| **Ant HOA 3mm** | FAZ | 0,163 | 0,159 | 0,851152416 |
| **OPD 6** | SCP-Whole | -0,123 | 0,288 | 0,887510204 |
| **OPD 6** | SCP-fovea | 0,037 | 0,748 | 0,939130435 |
| **OPD 6** | SCP- parafovea | -0,122 | | 0,887510204 |

| | | | | |
|---|---|---|---|---|
| **OPD 6** | SCP-perifovea | -0,173 | 0,295 | 0,851152416 |
| **OPD 6** | DCP-Whole | -0,077 | 0,135 | 0,892518337 |
| **OPD 6** | DCP-fovea | -0,019 | 0,506 | 0,962951576 |
| **OPD 6** | DCP-parafovea | -0,098 | 0,87 | 0,887510204 |
| **OPD 6** | DCP-perifovea | -0,093 | 0,399 | 0,887510204 |
| **OPD 6** | FAZ | 0,092 | 0,424 | 0,887510204 |
| | | | 0,429 | |
| **SR6** | SCP-Whole | 0,138 | 0,235 | 0,887510204 |
| **SR6** | SCP-fovea | 0,023 | 0,842 | 0,957368833 |
| **SR6** | SCP- parafovea | 0,157 | 0,175 | 0,876955017 |
| **SR6** | SCP-perifovea | 0,2 | 0,083 | 0,83 |
| **SR6** | DCP-Whole | 0,099 | 0,397 | 0,887510204 |
| **SR6** | DCP-fovea | 0,087 | 0,457 | 0,89088 |
| **SR6** | DCP-parafovea | 0,049 | 0,673 | 0,915127479 |
| **SR6** | DCP-perifovea | 0,114 | 0,328 | 0,887510204 |
| **SR6** | FAZ | -0,169 | 0,144 | 0,851152416 |

RMS: root mean square; HOA: higher-order aberrations; SA: spherical aberration; OPD: optical path difference; SR: Strehl ratio; SCP: superficial capillary plexus; DCP: deep capillary plexus; FAZ: foveal avascular zone.

*P*<0.05 is statistically significant. FDR <0.05 is statistically significant.
